# Bilateral facial paralysis associated with leptospirosis

**DOI:** 10.1590/0100-3984.2017.0050

**Published:** 2018

**Authors:** Aston Midon, Fabiana Batista Corrêa, Raphael Doyle Maia, Aline Gasparini Sampaio, Marcos Rosa Júnior

**Affiliations:** 1 Universidade Federal do Espírito Santo (UFES), Vitória, ES, Brazil.

*Dear Editor,*

**Figure 1:**
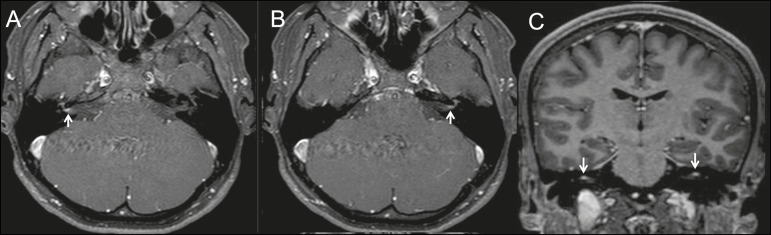
Magnetic resonance imaging, T1-weighted acquisition after administration of gadolinium in the axial plane (A,B) and coronal plane (C), showing linear contrast enhancement of the facial nerves within the internal auditory channels.

A 26-year-old male with a history of contact with rodents was admitted with headache, myalgia, arthralgia, high fever, abdominal pain, vomiting, and hepatosplenomegaly, evolving to jaundice, conjunctival suffusion, edema of the extremities, and pleural effusion. The serological investigation showed positivity for immunoglobulin M antibodies against *Leptospira* spp., and the patient improved after specific antibiotic therapy. However, he presented bilateral facial paralysis on day 11 after symptom onset. Magnetic resonance imaging with gadolinium contrast showed linear contrast enhancement in the cisternal, tympanic, labyrinthine, and geniculate ganglia of the facial nerves (Figure 1). After treatment with prednisone, there was complete improvement of the condition.

Leptospirosis is an acute febrile illness, common in tropical countries, caused by *Leptospira* interrogans. Its incidence is related to precarious living conditions, which favor the proliferation of rodents, the main vectors of the disease. Infected rodents eliminate the spirochetes in their urine, which contaminates the rainwater, exposing the human population to the agent, especially during flooding^(1)^.

Neurological symptoms can comprise the clinical spectrum of the disease and are present in 12% to 40% of cases. There have been few reported cases of leptospirosis accompanied by facial paralysis, and the occurrence of bilateral facial paralysis in leptospirosis patients is even more uncommon^(2,3)^.

Bilateral facial paralysis is quite rare and is usually related to systemic diseases. Unlike the unilateral form, bilateral facial paralysis is idiopathic in only 20% of cases^(4)^. The differential diagnosis is broad^(4-7)^, the main causes being Lyme disease (in 36.0% of cases), Guillain-Barré syndrome (in 5.0%), trauma (in 4.0%), sarcoidosis (in 0.9%), and AIDS (in 0.9%).

The diagnosis of bilateral facial paralysis depends on the patient history, which will guide the subsequent investigation. In the investigation of such patients, the priority is to diagnose life-threatening conditions, such as leukemia and Guillain-Barré syndrome. In addition to laboratory tests—such as a complete blood count, treponemal tests, serology for HIV, serology for Lyme disease, and lumbar puncture, as well as quantification of the blood glucose level, the erythrocyte sedimentation rate, and antinuclear antibodies—and magnetic resonance imaging studies of the brain for the investigation of the involvement of other cranial nerves or other associated findings—a complete physical examination should be performed^(7)^. Because leptospirosis is a common entity during times of flooding in Brazil, we must also consider the disease in patients who develop bilateral facial paralysis and we should therefore include specific tests for its diagnosis^(1)^.

In the case reported here, there was bilateral facial paralysis in the course of hemorrhagic fever with jaundice and, more specifically, on day 11 after symptom onset, which is consistent with other reports in the literature, in which facial paralysis appeared during the immune phase of the disease. Although the pathogenesis of the condition is still not well known, it is believed that *Leptospira* spp. can cause systemic vasculitis and activate the formation of immunocomplexes^(1)^. However, a specific causal relationship between leptospirosis and facial paralysis has yet to be established, and the description of new cases is therefore fundamental for a better understanding of the relationship between the two entities. Further studies on the subject should be encouraged.
